# Vitamin D Receptor (VDR) Gene Polymorphisms and High-Turnover Renal Osteodystrophy or Secondary Hyperparathyroidism in End-Stage Renal Disease: A Systematic Review

**DOI:** 10.7759/cureus.64925

**Published:** 2024-07-19

**Authors:** Tanya Sinha, Muhammad Muaz Mushtaq, Husnain Ali, Maryyam Liaqat, Maham Mushtaq, Muhammad Ahmad Sarwar, Muhammad Asad Sarwer, Danyal Bakht, Rida Fatima, Syed Faqeer Hussain Bokhari

**Affiliations:** 1 Internal Medicine, Tribhuvan University, Kathmandu, NPL; 2 Medicine and Surgery, King Edward Medical University, Lahore, PAK; 3 Internal Medicine, King Edward Medical University, Lahore, PAK; 4 Medicine, Shaikh Khalifa Bin Zayed Al-Nahyan Medical and Dental College, Lahore, PAK; 5 Medicine, King Edward Medical University, Lahore, PAK; 6 Medicine and Surgery, Fatima Jinnah Medical University, Lahore, PAK; 7 Surgery, King Edward Medical University, Lahore, PAK

**Keywords:** vitamin d, pth, review, renal, end-stage kidney disease, ckd, osteodystrophy

## Abstract

Chronic kidney disease (CKD) and end-stage renal disease (ESRD) are often complicated by high-turnover renal osteodystrophy (HTRO) and secondary hyperparathyroidism (SHPT), characterized by disturbances in mineral metabolism and skeletal abnormalities. Genetic variations within the vitamin D receptor (VDR) gene, known as VDR gene polymorphisms, have been implicated in modulating the susceptibility to HTRO and SHPT. This systematic review aims to evaluate the existing literature on the association between VDR gene polymorphisms and the development of these complications in ESRD and hemodialysis patients. A comprehensive literature search across multiple databases was conducted, and studies investigating VDR gene polymorphisms and HTRO or SHPT in ESRD or hemodialysis patients were included. The included studies examined various VDR gene polymorphisms, such as BsmI, ApaI, TaqI, and FokI, and their associations with clinical outcomes like parathyroid hormone (PTH) levels, bone mineral density, and the development of SHPT or HTRO. The findings suggest that certain VDR gene polymorphisms, notably the ApaI "aa" genotype, BsmI "bb" genotype, TaqI "tt" genotype, and FokI variant, may contribute to the pathogenesis of SHPT and HTRO by affecting PTH levels, bone turnover markers, and vitamin D sensitivity. However, the studies had relatively small sample sizes and were conducted in different populations, limiting generalizability. Further larger-scale studies, functional investigations, and exploration of gene-environment interactions are warranted to elucidate the underlying mechanisms and facilitate personalized treatment approaches for CKD and ESRD patients with mineral and bone disorders.

## Introduction and background

Chronic kidney disease (CKD), the seventh-most common cause of worldwide mortality, represents a significant global health burden, affecting millions of individuals and necessitating long-term management strategies to mitigate associated complications [1]. Among the myriad complications accompanying CKD, high-turnover renal osteodystrophy (HTRO) and secondary hyperparathyroidism (SHPT) stand out as critical concerns, particularly in end-stage renal disease (ESRD) and hemodialysis patients [2]. HTRO and SHPT are characterized by disturbances in mineral metabolism, skeletal abnormalities, and imbalanced parathyroid hormone (PTH) secretion, leading to heightened morbidity and mortality rates in affected populations [2]. Understanding the genetic determinants underlying the development and progression of these complications is paramount in improving patient outcomes and refining therapeutic approaches.

The vitamin D receptor (VDR) gene, located on chromosome 12q, plays a pivotal role in regulating calcium and phosphorus homeostasis, bone metabolism, and calcitriol synthesis, the active form of vitamin D [3]. Genetic variations within the VDR gene, known as VDR gene polymorphisms, have been implicated in modulating individual susceptibility to HTRO and SHPT in ESRD and hemodialysis patients [4,5]. These polymorphisms encompass single nucleotide polymorphisms (SNPs) and other genetic alterations that may influence VDR expression, function, or responsiveness to vitamin D analogs and other therapeutic interventions. Notable mentions in VDR polymorphism include FokI (rs2228570), TaqI (rs731236), BsmI (rs1544410), and ApaI (rs7975232) [5]. The significance of investigating VDR gene polymorphisms in the context of ESRD and hemodialysis lies in their potential to serve as genetic markers for disease risk stratification, prognostication, and personalized treatment approaches. Identifying specific VDR polymorphisms associated with increased susceptibility to HTRO and SHPT may aid clinicians in tailoring preventive measures and therapeutic regimens, thereby optimizing patient care and minimizing adverse outcomes [6,7]. Moreover, elucidating the molecular mechanisms underlying these associations could pave the way for developing novel targeted therapies that are aimed at modulating VDR activity and mitigating mineral and bone disorders in CKD patients.

The primary objective of this systematic review is to comprehensively evaluate the existing literature on the association between VDR gene polymorphisms and the development of HTRO or SHPT in ESRD and hemodialysis patients. By systematically synthesizing and analyzing data from relevant studies, we aim to elucidate the strength and consistency of these associations, identify potential sources of heterogeneity or bias, and elucidate gaps in current knowledge. Additionally, this review seeks to provide insights into the clinical implications of VDR gene polymorphisms in risk prediction, diagnostic algorithms, and therapeutic decision-making for HTRO and SHPT management in CKD populations.

## Review

Materials and methods

This systematic review adheres to the Preferred Reporting Items for Systematic Reviews and Meta-Analyses (PRISMA) guidelines, ensuring a rigorous and comprehensive evaluation of studies investigating the association between VDR gene polymorphisms and the development of HTRO or SHPT in ESRD or hemodialysis patients.

Search Strategy

A systematic search strategy was implemented across prominent electronic databases, including PubMed, Hinari, and Cochrane. The search strategy utilized a combination of Medical Subject Headings (MeSH) terms and keywords, such as VDR gene polymorphisms, HTRO, secondary hyperparathyroidism, ESRD, and hemodialysis. Boolean operators (AND, OR) were applied to refine the search and identify the studies meeting predefined inclusion criteria.

Eligibility Criteria

Rigorous eligibility criteria were meticulously defined to ensure the inclusion of studies of utmost quality and relevance. Studies selected for consideration had to investigate the association between VDR gene polymorphisms and the development of HTRO or SHPT in ESRD or hemodialysis patients. Additionally, the selected studies had to be published in peer-reviewed journals within the specified temporal scope from the inception of pertinent databases to February 2024. Studies lacking adequate data on VDR gene polymorphisms or not focusing on HTRO or SHPT in ESRD or hemodialysis patients were excluded. Furthermore, studies conducted solely on animal models were excluded to prioritize human-relevant findings. Lastly, studies published in languages other than English or those lacking full-text availability were excluded to ensure accessibility and comprehensibility for the systematic review process.

Data Extraction and Synthesis

Two independent reviewers conducted the initial screening of titles and abstracts, followed by a detailed assessment of the full texts to ensure adherence to the inclusion criteria. Any discrepancies between reviewers were resolved through discussion and, if necessary, consultation with a third reviewer. Relevant data, including study characteristics, sample size, VDR gene polymorphisms studied, and outcomes related to HTRO or SHPT, were systematically extracted using a predefined data extraction form.

Data Analysis

A narrative synthesis approach was employed due to anticipated heterogeneity in study designs, patient populations, and outcome measures. Key themes and patterns related to the association between VDR gene polymorphisms and the development of HTRO or SHPT are identified and presented. This method ensures a comprehensive and transparent evaluation of the existing literature.

Results

Study Selection Process

**Figure 1:**
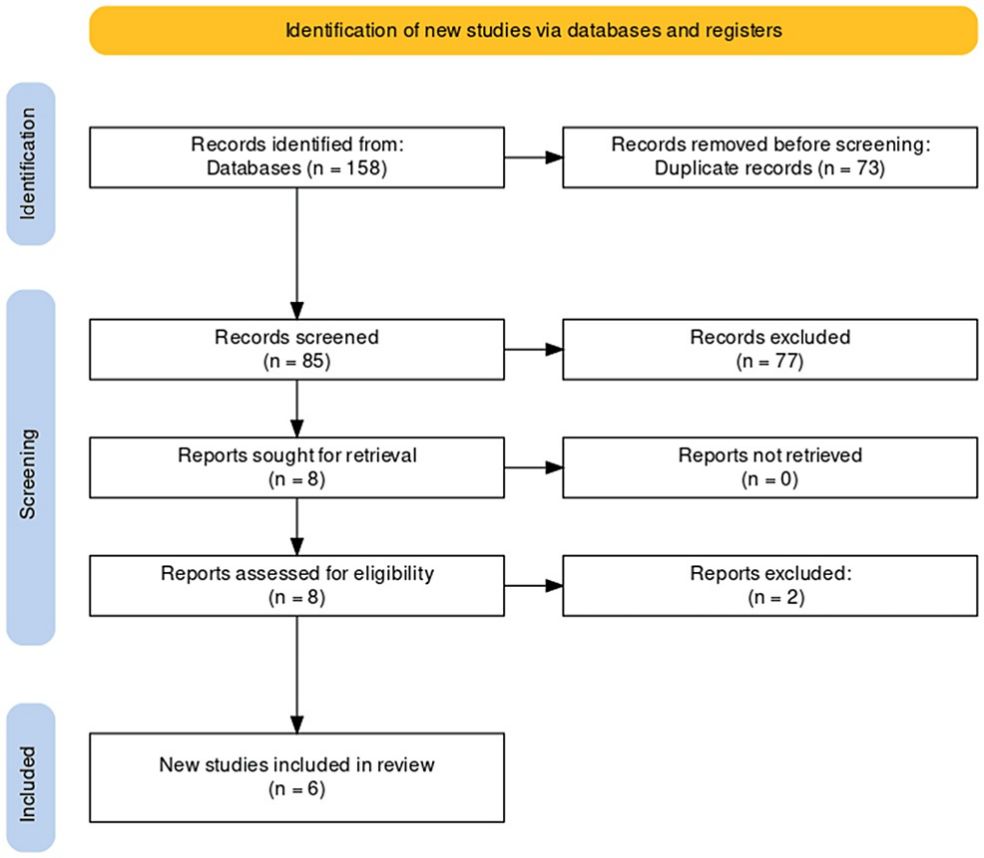
PRISMA diagram illustrating the study selection process PRISMA: Preferred Reporting Items for Systematic Reviews and Meta-Analyses.

The study selection process was meticulously conducted to ensure transparency according to the PRISMA guidelines. Initially, a comprehensive search across prominent electronic databases yielded 158 potentially relevant studies. After removing 73 duplicates, a refined pool of 85 unique studies was obtained. Subsequently, titles and abstracts were screened, resulting in the exclusion of 77 records that did not meet predefined relevance criteria. The full texts of the remaining eight articles were meticulously evaluated to assess their alignment with stringent inclusion criteria. Following this rigorous selection process, six studies were identified as meeting the eligibility criteria and were deemed suitable for inclusion in the systematic review. The study selection process is visually depicted in the PRISMA flowchart, which illustrates the sequential stages of study identification, screening, eligibility assessment, and final inclusion (Figure 1).

Study Characteristics

The included studies were mostly cross-sectional (n = 5) with one case-control study. The sample sizes ranged from 46 to 904 participants, with the majority being hemodialysis or ESRD patients. The studies were conducted in different countries, including Japan, Spain, Italy, Iran, and China, providing a diverse representation of populations. The primary focus was on investigating the association between various VDR gene polymorphisms, such as BsmI, ApaI, TaqI, and FokI, and clinical outcomes such as PTH levels, bone mineral density, and the development of SHPT or HTRO. The studies examined different combinations of VDR gene polymorphisms and their potential impact on these clinical outcomes in the context of CKD or ESRD (Table 1).

**Table 1:** Study characteristics and summary of the main findings of the included studies PTH: Parathyroid hormone; CRF: Chronic renal failure; ESRD: End-stage renal disease; PTH: Parathyroid hormone; SHPT: Secondary hyperparathyroidism; HTRO: High-turnover renal osteodystrophy; iPTH: Intact parathyroid hormone; VDR: Vitamin D receptor.

Authors	Year	Country	Study type	Sample size	VDR genes discussed	Biomarkers	Main findings
Yokoyama et al. [8]	1998	Japan	Cross-sectional	129 hemodialysis patients	BsmI, ApaI	PTH, osteocalcin	The study found that the frequency of the BsmI bb genotype was very high (80.6%) in this Japanese population, making it less suitable for analysis. However, ApaI polymorphisms showed that ApaI aa genotype was associated with significantly higher serum PTH and intact osteocalcin levels compared to AA/Aa genotypes, suggesting this variant may promote SHPT and high bone turnover.
Marco et al. [9]	1999	Spain	Cross-sectional	248 pre-dialysis CRF patients	BsmI	PTH, calcitriol	The study showed that in pre-dialysis CRF patients, those with the bb and Bb VDR genotypes had faster progression of SHPT and lower calcitriol levels compared to the BB genotype, suggesting this variant promotes more severe disease.
Giannini et al. [10]	2002	Italy	Cross-sectional	69 renal transplant recipients	BsmI	PTH, bone density, bone turnover markers	The study found that the bb VDR genotype was associated with higher PTH levels and lower spinal bone density after renal transplantation, suggesting this variant promotes more severe SHPT.
Pourfarzam et al. [11]	2014	Iran	Cross-sectional	90 hemodialysis patients	BsmI, TaqI	PTH	Patients with the TaqI tt or BsmI BB genotypes tended to have higher serum PTH levels, suggesting these variants may be linked to more severe SHPT.
Ghorbanihagjo et al. [12]	2014	Iran	Cross-sectional	46 hemodialysis patients, 43 controls	FokI, ApaI	Fetuin-A, vitamin D, PTH	The study examined the association between VDR gene FokI and ApaI polymorphisms and serum levels of fetuin-A, vitamin D, and intact PTH in Iranian hemodialysis patients. Patients with FokI variant had higher PTH levels. Patients with ApaI variant had lower vitamin D levels compared to controls, suggesting these variants may promote vascular calcification risk.
Wang et al. [13]	2016	China	Case-control	452 ESRD patients, 904 controls	TaqI, ApaI	PTH	The study found that the ApaI C allele was associated with increased ESRD risk and higher iPTH levels in Chinese patients, suggesting it may promote SHPT and HTRO. No association was seen for the TaqI variant.

Quality Assessment

The quality assessment of the included studies was performed using Newcastle-Ottawa Quality Assessment Scales. Three studies were of high quality, two were of moderate quality, and one study was of good quality (Table 2).

**Table 2:** Quality assessment of included studies using the Newcastle-Ottawa Quality Assessment Scales

Study	Selection (Max. 4*)	Comparability (Max. 2*)	Outcome/Exposure (Max. 3*)	Total score
Yokoyama et al. [8]	***	**	**	7
Marco et al. [9]	****	**	***	9
Giannini et al. [10]	****	**	***	9
Pourfarzam et al. [11]	***	**	***	8
Ghorbanihagjo et al. [12]	***	**	**	7
Wang et al. [13]	****	**	***	9

Discussion

VDR is a nuclear transcription factor that mediates the biological actions of the active form of vitamin D, calcitriol [14]. VDR plays a crucial role in regulating various physiological processes, including calcium and phosphate homeostasis, bone metabolism, and cellular proliferation and differentiation. The VDR gene is highly polymorphic, with several genetic variations or SNPs identified, such as BsmI, ApaI, TaqI, and FokI [5]. These VDR gene polymorphisms have been studied extensively in relation to various diseases and conditions, including CKD and ESRD. In CKD and ESRD patients, the development of SHPT and HTRO is a common complication [15]. SHPT is characterized by elevated PTH levels, which can lead to bone disease, vascular calcification, and other adverse outcomes [15]. The studies included in this systematic review aimed to investigate the association between VDR gene polymorphisms and the development of SHPT or HTRO in ESRD or hemodialysis patients. The findings from these studies suggest that certain VDR gene polymorphisms may contribute to the pathogenesis of these complications.

Several studies found that the ApaI "aa" genotype was associated with higher PTH levels, increased bone turnover markers (such as intact osteocalcin), and a higher risk of secondary hyperparathyroidism in ESRD and hemodialysis patients [8,12,13]. The ApaI "C" allele was also linked to an increased risk of ESRD and higher PTH levels in the Chinese population [13]. The BsmI "bb" genotype was associated with higher PTH levels, lower calcitriol levels, and faster progression of SHPT in pre-dialysis CKD patients and renal transplant recipients [9,10]. Additionally, the TaqI "tt" genotype and BsmI "BB" genotype also showed a trend toward higher PTH levels in Iranian hemodialysis patients [11]. The FokI variant was found to be more frequent in hemodialysis patients compared to controls and was associated with higher PTH levels, suggesting a potential role in the development of SHPT [12]. These findings highlight the potential impact of VDR gene polymorphisms on the dysregulation of mineral metabolism and bone homeostasis in CKD and ESRD patients. The presence of specific VDR variants may contribute to the development of SHPT and HTRO by affecting the sensitivity and responsiveness to vitamin D and its regulatory mechanisms.

It is important to note that the studies included in this systematic review had relatively small sample sizes and were conducted in different populations, which may affect the generalizability of the findings. Additionally, the functional mechanisms by which these VDR gene polymorphisms influence the development of SHPT and HTRO are not fully understood and may involve complex interactions with other genetic and environmental factors [16]. Further investigation through larger prospective studies involving diverse populations is imperative to delve deeper into the correlations between VDR gene polymorphisms and the onset of SHPT and HTRO in patients with CKD and ESRD. Additionally, conducting functional studies to scrutinize the molecular mechanisms influenced by specific VDR gene polymorphisms in vitamin D signaling, calcium and phosphate homeostasis, and PTH regulation would yield invaluable insights into the underlying pathogenesis of these complications. Furthermore, exploring potential interactions between VDR gene polymorphisms and other genetic variations, alongside environmental factors like dietary habits, sun exposure, and medication usage, is crucial for unraveling the intricate interplay of risk factors [17]. Such endeavors may pave the way for personalized treatment approaches tailored to an individual VDR genotype, thereby optimizing the management of SHPT and bone disease in CKD and ESRD patients. Moreover, the development of innovative therapeutic strategies targeting the VDR or its downstream signaling pathways holds promise in enhancing the management of mineral and bone disorders in this patient population.

## Conclusions

This systematic review highlights the potential impact of VDR gene polymorphisms on the development of SHPT and HTRO in ESRD and hemodialysis patients. Variants such as ApaI, BsmI, TaqI, and FokI have been associated with dysregulated PTH levels, bone turnover markers, and vitamin D sensitivity. However, further research with larger sample sizes and diverse populations is crucial to establish stronger associations and unravel the underlying mechanisms. Functional studies and exploration of gene-environment interactions may pave the way for personalized treatment strategies tailored to individual VDR genotype, optimizing the management of mineral and bone disorders in CKD and ESRD.
